# The impact of COVID-19 on the prognosis of deep vein thrombosis following anticoagulation treatment: a two-year single-center retrospective cohort study

**DOI:** 10.1186/s12890-024-03036-3

**Published:** 2024-04-26

**Authors:** Qi Wang, Jiajun Wu, Pengfei Zhang, Xu Ma

**Affiliations:** 1 Department of emergency, School of Medicine, Shanghai Ninth People’s Hospital, Shanghai JiaoTong University, No. 639 Zhizuoju Road, Shanghai, China; 2 Department of Ultrasound, Shanghai Pulmonary Hospital, School of Medicine, Tongji University, No. 507 Zhengmin Road, Shanghai, 200433 China; 3 Department of Radiology, Shanghai Pulmonary Hospital, School of Medicine, Tongji University, No. 507 Zhengmin Road, Shanghai, 200433 China

**Keywords:** Deep vein thrombosis, Coronavirus disease 2019, Anticoagulation treatment, Evaluation of response

## Abstract

**Background:**

Coronavirus disease 2019 (COVID-19) has been proved as a significant risk factor for deep vein thrombosis (DVT) after several waves of pandemic. This study aims to further investigate impact of COVID-19 on prognosis of DVT following anticoagulation treatment.

**Methods:**

A total of 197 patients with initially detected DVT and meanwhile accomplishing at least 3 months anticoagulation treatment were identified from our hospital between January 2021 and December 2022. DVT characteristics, clinical data, and exposure to COVID-19 were recorded for multivariable logistic regression analysis to identify DVT aggravation related risk factors. Propensity score matching (PSM) was used to balance baseline covariates. Kaplan–Meier curves and Log-Rank test were performed to exhibit distribution of DVT aggravation among different subgroups.

**Results:**

In 2022, patients exhibited higher incidence rates of DVT aggravation compared to those in 2021 (HR:2.311, *P* = 0.0018). The exposure to COVID-19, increased red blood cell count, increased D-dimer level and reduced prothrombin time were found to be associated with DVT aggravation (*P* < 0.0001, *P* = 0.014, *P* < 0.001, *P* = 0.024), with only exposure to COVID-19 showing a significant difference between two years (2022:59/102, 57.84%, 2021:7/88, 7.37%, *P* < 0.001). In PSM-matched cohorts, the risk for DVT aggravation was 3.182 times higher in COVID-19 group compared to the control group (*P* < 0.0001). Exposure to COVID-19 increased the risk of DVT aggravation among patients who completed three months anticoagulant therapy (HR: 5.667, *P* < 0.0001), but did not increase incidence rate among patients who completed more than three months anticoagulant therapy (HR:1.198, *P* = 0.683). For patients with distal DVT, COVID-19 was associated with a significant increased risk of DVT recurrence (HR:4.203, *P* < 0.0001). Regarding principal diagnoses, incidence rate of DVT aggravation was significantly higher in COVID-19 group compared to the control group (Advanced lung cancer: *P* = 0.011, surgical history: *P* = 0.0365, benign lung diseases: *P* = 0.0418).

**Conclusions:**

Our study reveals an increased risk of DVT aggravation following COVID-19 during anticoagulation treatment, particularly among patients with distal DVT or those who have completed only three months anticoagulant therapy. Adverse effects of COVID-19 on DVT prognosis were observed across various benign and malignant respiratory diseases. Additionally, extended-term anticoagulant therapy was identified as an effective approach to enhance DVT control among patients with COVID-19.

## Background

Deep vein thrombosis (DVT) is a multifactorial disease. The classical clinical signs include pain and swelling in the lower limb, but the symptoms of DVT are hard to detect in most cases [1, 2]. Despite its relatively low incidence rate, acute DVT can lead to pulmonary embolism (PE), while nearly half of chronic DVT cases progress to the post-thrombotic syndrome (PTS) [3–5]. These complications not only significantly impact quality of life but also can pose life-threatening instances. Therefore, it is crucial to implement appropriate treatment and timely preventive measures for DVT. Numerous studies have identified various risk factors associated with DVT, including body mass index (BMI) ≥ 30, tumor status, anti-tumor treatments, abnormal coagulation function, tuberculosis, and acute trauma, among others [6–11]. In recent years, coronavirus disease 2019 (COVID-19) has been confirmed to be an important risk factor for DVT [12–14]. Nevertheless, the potential impact of COVID-19 on the prognosis of DVT during treatment remains a topic requiring further exploration after several pandemic waves.

Numerous trails and retrospective studies have been designed on the risk of DVT after COVID-19, but limited researches have focused on the impact of COVID-19 on prognosis of DVT after anticoagulation treatment [15–18]. Therefore, our study aimed to evaluate the efficacy and safety of anticoagulant therapy for DVT in patients with various respiratory benign diseases and malignant diseases, both with and without concurrent COVID-19.

## Methods and materials

### Study population and clinical characteristics

We retrospectively analyzed a total of 4,376 duplex ultrasound scan (DUS) reports of lower limb deep veins and corresponding clinical data in our hospital between January 2021 and December 2022. The DUS was performed following the whole leg compressive ultrasound protocol, including bilateral examination of common femoral, femoral, popliteal, and deep calf veins in accordance. DVT was defined as a visible intraluminal content in the noncompressible or partially compressible veins. This study specifically enrolled patients who were diagnosed with DVT for the first time and routinely received DUS at least once a month. All enrolled patients underwent telephone follow-up surveys, which primarily focused on their exposure history to COVID-19, selection of anticoagulant drugs, duration of anticoagulant therapy, and occurrence of hemorrhagic events. The exclusion criteria included: (1) DUS follow-up time being less than three months; (2) absence of any antithrombotic therapy; and (3) duration of anticoagulant therapy being less than 3 months. We recorded the baseline data, clinical characteristics and DVT attributes of enrolled patients for further analysis. Risk scoring results (Paudua score for medical patients, Caprini score for surgical patients, and Khorana score for tumor patients) for venous thromboembolism (VTE) were categorized into three risk grades as follows: grade 0 (Paudua score = 0, Caprini score = 0, and Khorana score = 0), grade 1 (Paudua score < 4, Caprini score < 3, and Khorana score < 3), and grade 2 (Paudua score ≥ 4, Caprini score ≥ 3, and Khorana score ≥ 3) [16, 19–21]. The exposure history of COVID-19 was confirmed if COVID-19 antigen test or PCR test was positive within 6 months before or after diagnosis of DVT. Currently received treatment referred to the on-going treatment within two weeks from the date as DVT was diagnosed. All enrolled patients with DVT underwent chest computered tomograhy angiography (CTA) to exclude PE.

The dosage of anticoagulants in the study were presented as follows: (1) rivaroxaban, 15 mg twice daily in the first 3 weeks, followed by 20 mg once daily; (2) edoxaban, 60 mg once daily; (3) enoxaparine, 100 AXalU per kilogram every 12 h in first week, followed 100 AXalU per kilogram once daily. Dose reduction was specified basing on the results of renal function, platelet count, and level of D-dimer (DD).

Evaluation of anticoagulation treatment was based on the results of follow-up DUS reports: (1) unchanged location and length of DVT was defined as stable condition; (2) reduced length of DVT or lower level from first detected location of DVT was defined as DVT remission; (3) increased length and upper level of DVT, or new detected location of DVT was defined as DVT aggravation. The stable DVT and DVT remission were both identified as the control of DVT. All enrolled patients were followed until death or the last follow up, and the endpoint of DVT was defined as the latest result obtained from DUS. Level of DD was also recorded for evaluation of anticoagulation treatments.

### Statistical analysis

The enrolled patients were divided into two groups (2021 and 2022) based on the earliest date of DVT diagnosis. Baseline data differences between the two groups were analyzed using t-test and chi-square test. Kaplan-Meier cumulative risk curves were presented to illustrate the temporal distribution of DVT aggravation from the earliest diagnosed date to the endpoint. All enrolled patients were divided into the responding group (DVT remission) and the non-responders (DVT stabilization or aggravation). A multivariable logistic regression model was employed to evaluate the related risk factors influencing anticoagulation treatment efficacy.

We conducted the propensity score–matched (PSM) analysis to investigate the impact of COVID-19 on the prognosis of DVT after at least 3 months of anticoagulation treatment. Therefore, all subjects were divided into two groups: the control group and COVID-19 group. The matching ratio between COVID-19 group and the control group was 1:2, using a nearest neighbor matching algorithm without replacement with distances determined by logistic regression. The matching covariates included age, diagnosis, and risk grades of DVT. Nearest-neighbor matching was performed with a match tolerance of 0.2 units of the pooled estimate of the common standard deviation of the logits of the propensity scores. The difference in DVT aggravation between these two cohorts was analyzed using Kaplan–Meier curves and the Log-Rank test.

In order to verify how COVID-19 influences DVT prognosis under different conditions within this 1:2 matched PSM set, we further divided these matched subjects into groups based on current principal diagnosis, duration of anticoagulation therapy, and type of DVT. Subgroup analyses were conducted to present the progression of DVT over time using Kaplan-Meier cumulative risk curves.

All statistical analysis were performed by SPSS version 22.0 (IBM Corp., Armonk, NY, USA). *P* value of < 0.05 was considered statistically significant.

## Results

### Demographic characteristics

**Fig. 1:**
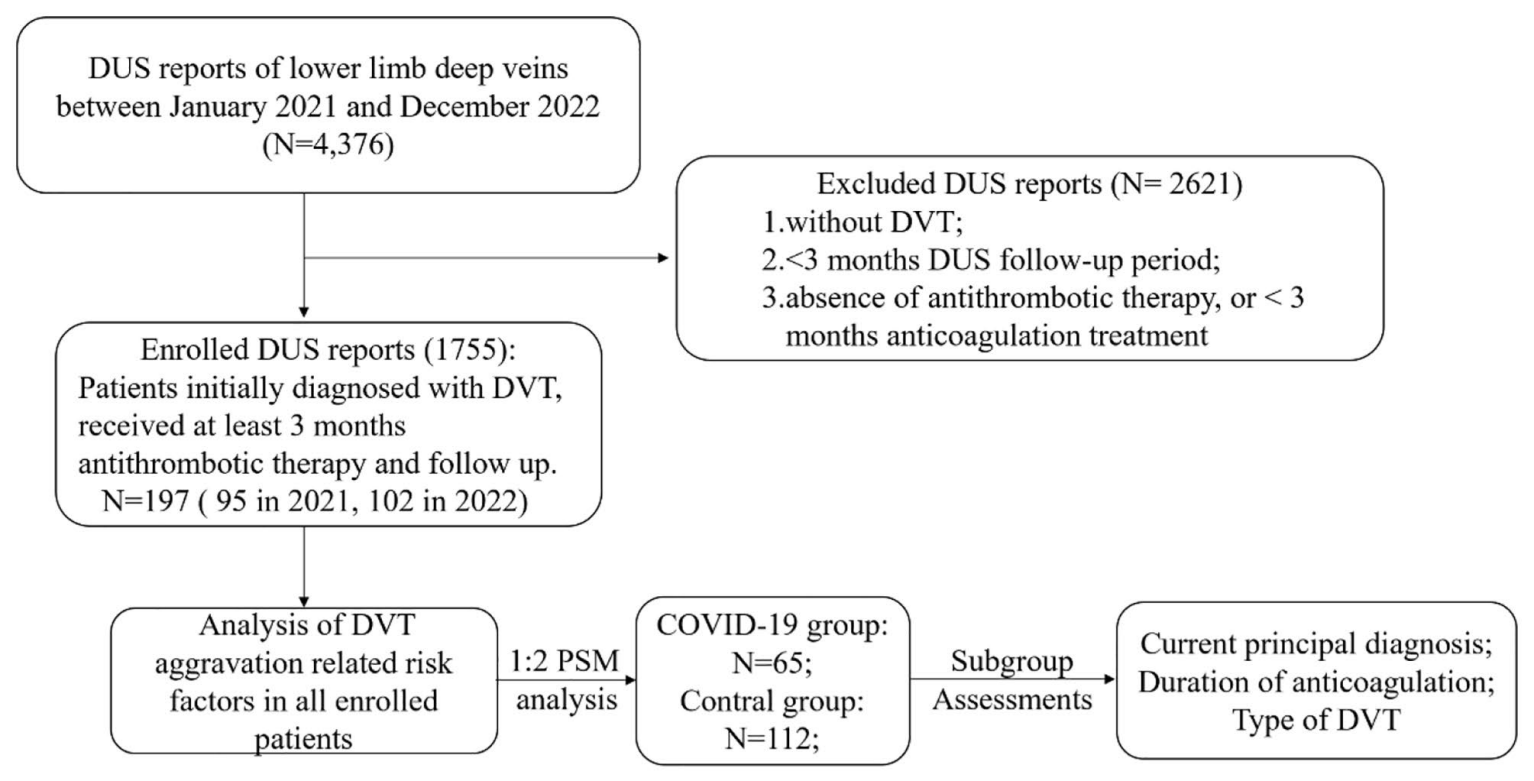
Flow chart

**Fig. 2:**
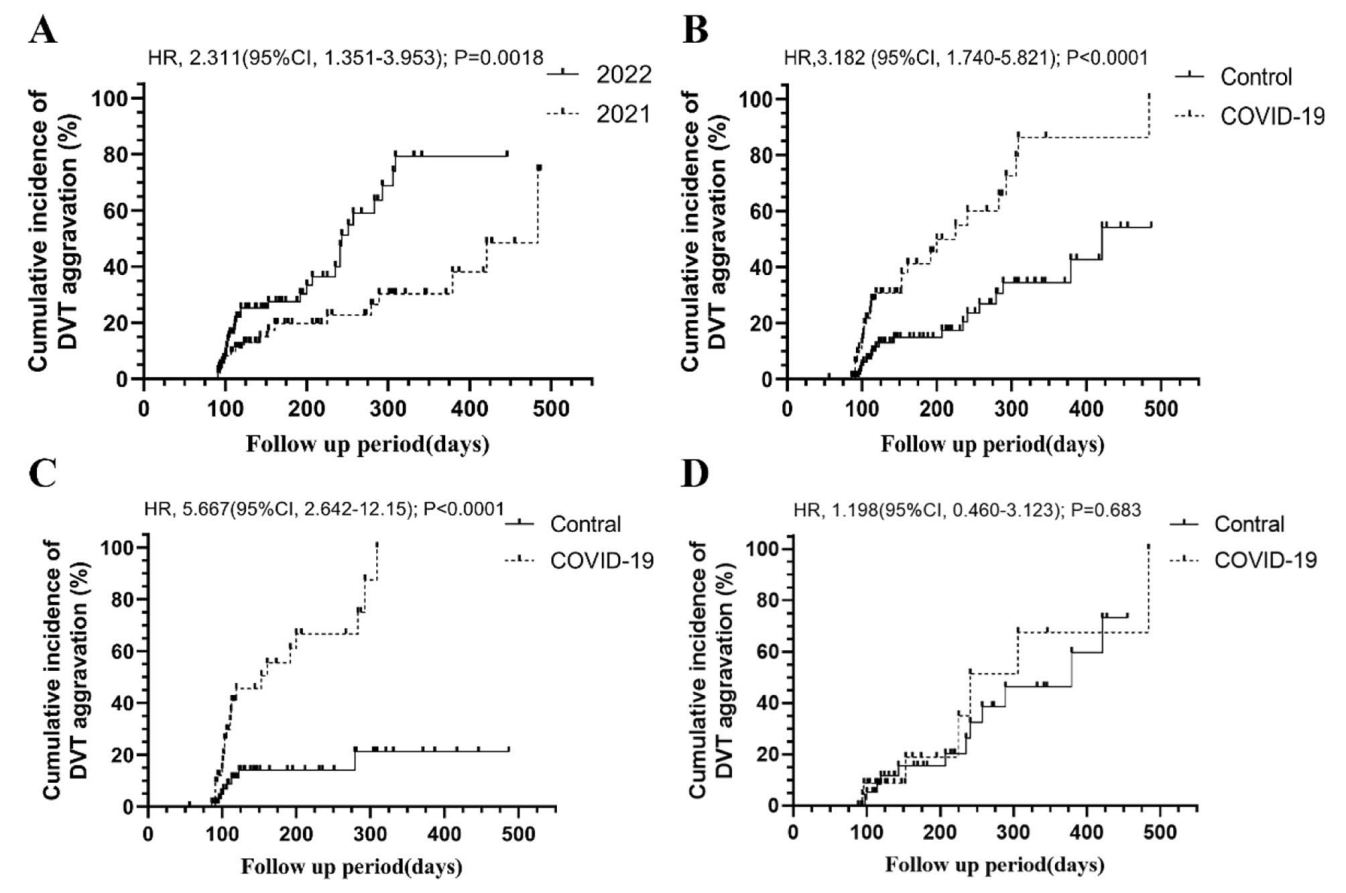
Cumulative incidence of DVT aggravation in different groups. **(A)** The cumulative incidence of DVT aggravation between patients in 2022 and those in 2021; **(B)** In the 1:2 PSM cohorts, the relative risk for DVT aggravation between COVID-19 group and the control group; **(C)** In the 1:2 PSM cohorts, the risk incidences for DVT aggravation between COVID-19 group and the control group at C among patients who completed only 3 months anticoagulant therapy; **(D).** In the 1:2 PSM cohorts, no significant difference in the DVT control rate between the COVID-19 group and the control group at D among patients who received more than three months of anticoagulant therapy

In the present study, we identified 197 patients who were initially diagnosed with DVT, received treatment and underwent a follow-up of at least 3 months at Shanghai Pulmonary Hospital from 2021 to 2022 (Fig. 1). The baseline demographics and clinical characteristics of enrolled patients are presented in Table 1. Patients’ clinical data indicated minimal disparities between the years 2021 and 2022. Despite the longer median follow-up duration in 2021 compared to that in 2022 (*P* < 0.0001), the control rate of DVT was higher in 2021 (76/95, 80.00%) than in 2022 (67/102, 65.69%, *P* = 0.005). Moreover, there was a significantly greater proportion of patients exposed to COVID-19 in 2022 (59/102,57.84%) than in 2021 (7/88, 7.37%, *P* < 0.001). There were more patients with low levels of platelet count (PLT) (27.5% VS 9.2%, *P* = 0.016) in 2022 compared with 2021. In addition, no significant differences were observed regarding the remaining baseline demographics, features of DVT, and basic clinical characteristics. Besides, there were no significant differences in types of anticoagulants used between patients in 2021 (rivaroxaban, 83(87.4%); edoxaban, 7(7.4%); enoxaparine, 5(5.3%)) and patients in 2022 (rivaroxaban, 83(81.3%); edoxaban, 7(16.7%); enoxaparine, 2(2%)) (*P* = 0.074). Considering anticoagulation duration, there was also no significant difference between patients in 2021 (3 months, 61; extended duration, 34) and patients in 2022 (3 months, 62; extended duration, 40) (*P* = 0.62). One patient completely stopped rivaroxaban on account of massive hemoptysis in 2021. One patient with massive hemoptysis and one patient with intermittent epistaxis completely stopped rivaroxaban in 2022. The cumulative incidence of DVT aggravation indicated a higher risk for patients in 2022 compared to those in 2021 (HR:2.311, 95%CI: 1.351–3.953, *P* = 0.0018) (Fig. 2A).

**Table 1:** The baseline demographics and clinical characteristics between 2021 and 2022

Characteristics	2021	2022	*P* value
**Demographics**			
Gender (Male/Female)	57/38	65/37	0.591
Age (Mean), years	65	67	0.45
BMI (Mean)	24	23	0.63
DBP(M ± SD), mmHg	76.22 ± 9.26	75.75 ± 17.74	0.09
**DVT attributes**			
Type of DVT			
Unilateral distal Unilateral proximal Bilateral distal Bilateral proximal	36(37.9%) 26(27.4%) 23(24.2%) 10(10.5%)	43(42.2%) 25(24.5%) 25(24.5%) 9(8.8%)	0.528
Risk grade of DVT			
0 grade 1 grade 2 grade	4(4.2%) 57(60%) 34(35.8%)	3(2.9%) 53(52%) 46(45.1%)	0.398
PE			
None Periphery Central	59(62.1%) 7(7.4%) 29(30.5%)	60(58.8%) 8(7.8%) 34(33.4%)	0.894
Median follow up (Range), days	124 (96–421)	116.5 (91–446)	< 0.0001
Hemorrhage events			
None Minor Major	79 15 1	91 10 1	0.199
Therapeutic outcomes			
Stable condition Remission Aggravation	23(24.2%) 53(55.8%) 19(20%)	33(32.3%) 34(33.3%) 35(34.3%)	0.005
**Clinical characteristics**			
Diagnosis			0.319
Advanced lung cancer (SCC/adenocarcinoma/SCLC)	10/33/4	10/34/5	
Postoperative	10	11	
Benign lung diseases (None/Interstitial lung disease/Tuberculosis/ COPD/Pneumonia/Bronchiectasis/PAH)	2/4/8/3/13/6/2	7/5/5/10/5/6/4	
Ongoing treatment			0.197
Antitumor	47(49.5%)	57(55.9%)	
HREZ anti-infection hemostasis None	8(8.4%) 23(24.2%) 4(4.2%) 13(13.7%)	5(4.9%) 14(13.7%) 5(4.9%) 21(20.6%)	
Time from active ongoing treatment to DVT confirmed, months (< 6/6–12/>12)	83/9/3	83/7/12	0.067
COVID-19(None/Previously diagnosed within 6 months)	88/7	43/59	< 0.001
**Relevant blood biochemistry tests**			
RBC (< 4.3*10^12^/L / >4.3*10^12^/L)	56/39	60/42	1
WBC (< 9.5*10^12^/L / >9.5*10^12^/L)	72/23	79/23	0.867
PLT (< 125*10^9^/L / >125*10^9^/L)	8/87	22/80	0.016
ALT (< 49IU/L / >49IU/L)	81/14	92/10	0.29
CRE (< 97umol/L / >97umol/L)	91/4	93/9	0.2
D-dimer level ( Decreased / Increased )	66/29	67/35	0.571
PT (< 11s / >11s)	18/77	27/75	0.237

BMI, body mass index; DBP, diastolic blood pressure; DVT, deep vein thrombosis; PE, pulmonary embolism; SCC, squamous-cell carcinoma; SCLC, small cell lung cancer; HREZ, isonicotinic acid hydrazide, rifampicin, ethambutol, pyrazinamide; RBC, red blood cell count; WBC, white blood cell count; PLT, blood platelet count; ALT, alanine transaminase; CRE, creatinine; PT, prothrombin time.

### Assessment of risk factors associated with DVT

**Fig. 3:**
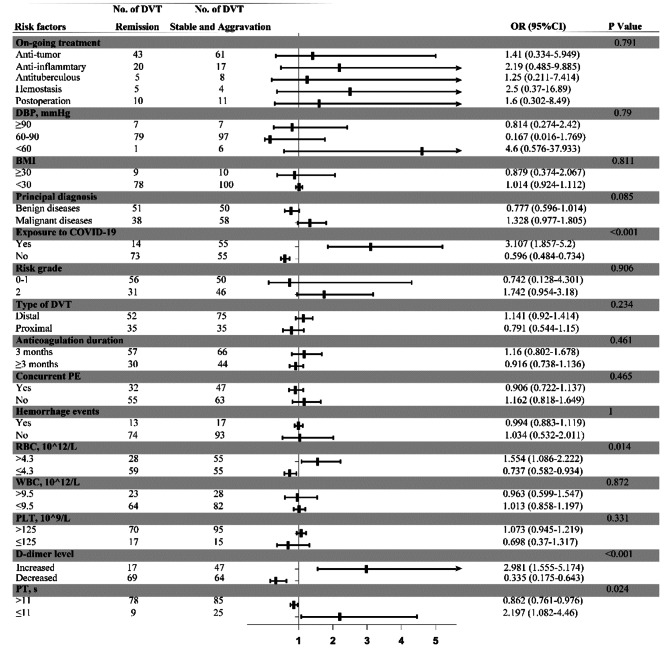
Forest plot illustrating the odds ratio of the composite outcome of DVT aggravation in patients achieving DVT remission compared to those experiencing DVT recurrence or aggravation. Abbreviations: DVT, deep vein thrombosis; OR, odds ratio; 95% CI, 95% credible interval; DBP, diastolic blood pressure; BMI, body mass index; PE, pulmonary embolism; RBC, red blood cell count; WBC, white blood cell count; PLT, blood platelet count; PT, prothrombin time

In the multivariate analysis of patient clinical background (principal diagnosis, BMI, diastolic blood pressure (DBP), COVID-19, and on-going treatment), DVT characteristics (type of DVT, risk grade, concurrent PE, hemorrhage events, anticoagulation duration), tumor factors (tumor staging and genetic mutation), and results from blood biochemistry tests (complete blood cell count, DD, and prothrombin time (PT)), COVID-19, increased red blood cell count (RBC), exposure to COVID-19, increased level of DD and reduced PT were identified as risk factors for DVT aggravation (Fig. 3).

Considering the potential relationship between abnormal renal function, liver disorders and DVT aggravation, we further assessed influence of elevated level of creatinine (CRE) and alanine transaminase (ALT). Both the abnormal level of CRE (OR:1.241, 95%CI:0.46–3.349, *P* = 0.35) and ALT (OR:0.747, 95%CI:0.318–1.758, *P* = 0.505) were not the risk factors for DVT aggravation.

### Comparison of DVT aggravation between COVID-19 group and control group in the unmatched and matched cohorts

Based on the differences of baseline characteristics between 2021 and 2022, we divided all enrolled patients into two groups: the COVID-19 group (69/197, 35.03%) and the control group (128/197, 64.97%). In the unmatched cohorts, no significant differences were observed in baseline demographics and clinical characteristics between two groups (Table 2). The incidence of DVT aggravation was higher in the COVID-19 group compared to the control group (31/69, 44.92% VS 23/128, 17.97%, *P* < 0.001), despite the shorter average follow-up duration (148 days vs. 176 days, *P* = 0.002). A significantly lower proportion of patients achieved DVT remission in the COVID-19 group (14/69, 20.29%) compared to the control group (73/128, 57.03%; *P* < 0.001).

**Table 2:** Comparison Between Baseline Clinicopathological Characteristics of COVID-19 VS control group

Characteristics	Unmatched cohort(*n* = 197)				1:2 Propensity score matching (*n* = 177)		
	COVID-19 (*n* = 69)	Contral (*n* = 128)	*P* value		COVID-19 (*n* = 65)	Contral (*n* = 112)	*P* value
**Demographics**							
Gender (Male/Female)	40/29	82/46	0.401		39/26	71/41	0.654
Age (Mean), years	66	66	0.619		66	66	0.491
BMI (Mean)	22	23	0.353		23	24	0.08
DBP(M ± SD), mmHg	74.71 ± 18.33	76.69 ± 11.52	0.135		78 ± 9.09	76.73 ± 12.06	0.356
**DVT attributes**							
Type of DVT (Unilateral distal/Unilateral proximal/Bilateral distal/Bilateral proximal )	28/15/18/8	51/36/30/11	0.739		26/15/18/6	42/32/28/10	0.884
Risk stratification of DVT (None/Low/High)	4/33/32	3/77/48	0.166		1/33/31	1/63/48	0.743
PE (None/Periphery/Central)	44/23/2	74/41/13	0.184		42/21/2	65/34/13	0.144
Mean follow up (Range), days	148.16 (91–484)	176.02(91–487)	0.002		149(91–484)	174.9(56–487)	0.004
Anticoagulant therapy (3 months/ Extended period)	45/24	78/50	0.554		42/23	69/43	0.69
Therapeutic outcomes (Stable condition/Remission/Aggravation)	24/14/31	32/73/23	< 0.001		23/13/29	28/64/20	< 0.001
**Clinical diagnosis**			0.235				0.34
Advanced lung cancer (SCC/adenocarcinoma/SCLC)	5/27/5	15/38/4			5/27/4	5/36/4	
Postoperative	8	16			8	16	
Benign lung diseases (None/Interstitial lung disease/Tuberculosis/ COPD/Pneumonia/Bronchiectasis/PAH)	4/2/3/7/2/3/3	5/7/10/6/15/9/3			2/2/3/7/2/2/3	5/6/9/6/14/8/3	
**Time from active ongoing treatment to DVT confirmed, months (< 6/6–12/>12)**	54/5/10	112/10/6	0.056		48/7/10	96/10/6	0.066
**Relevant blood biochemistry tests**							
RBC (< 4.3*10^12^/L / >4.3*10^12^/L)	37/32	78/50	0.364		34/31	72/40	0.152
WBC (< 9.5*10^12^/L / >9.5*10^12^/L)	58/11	95/33	0.153		52/13	79/33	0.214
PLT (< 125*10^9^/L / >125*10^9^/L)	11/58	19/109	0.838		8/57	18/94	0.66
D-dimer level (Decreased / Increased)	43/26	90/38	0.253		41/24	73/29	0.778
PT (< 11s / >11s)	13/56	21/107	0.695		11/54	17/95	0.832

BMI, body mass index; DBP, diastolic blood pressure; DVT, deep vein thrombosis; PE, pulmonary embolism; SCC, squamous-cell carcinoma; SCLC, small cell lung cancer; IVCT, intravenous chemotherapy; PD1, programmeddeath-1; TKI, tyrosine kinase inhibitor; RT, radiotherapy; HREZ, isonicotinic acid hydrazide, rifampicin, ethambutol, pyrazinamide; RBC, red blood cell count; WBC, white blood cell count; PLT, blood platelet count; PT, prothrombin time.

After implementing a 1:2 PSM based on the factors described in the methods section, we successfully matched 65 patients from the COVID-19 group with 112 patients from the control group (Table 2). In the 1:2 matched PSM set, the COVID-19 group was also associated with increased incidence rate of DVT aggravation compared to the control group (44.62% vs. 17.86%, *P* < 0.001), as well as reduced incidence rates of DVT remission (20% vs. 57.14%, *P* < 0.001). However, it is important to note that patients in the COVID-19 group had a significantly shorter mean follow-up time than those in the control group (149 vs. 174.9 days; *P* < 0.001). There were no significant differences in the remaining baseline characteristics between two groups, which was consistent with the results before matching (Table 2). The relative risk for DVT aggravation was found to be 3.182 times higher in COVID-19 group than in the control group (95%CI: 1.740–5.821, *P* < 0.0001), and the COVID-19 group (38/65, 58%) was 27% less likely to have an event of 6-month DVT remission compared to the control group (95/112, 85%, *P* < 0.0001) (Fig. 2B).

### Subgroup assessments

In the matched cohorts, results of Kaplan–Meier curves and the Log-Rank test further explained how COVID-19 impacts the prognosis of DVT during the anticoagulant therapy under various conditions.

Among patients who completed only 3 months anticoagulant therapy, the risk for DVT aggravation in COVID-19 group was 5.667 times higher than in the control group (95%CI: 2.642–12.15, *P* < 0.0001) (Fig. 2C). However, no significant difference in the DVT control rate at 6 and even 12 months between the COVID-19 group (6-month: 19/23, 83%, 12-month:8/23,35%) and the control group (6-month:37/43, 86%, 12-month:23/53%) among patients who received more than three months of anticoagulant therapy (HR:1.198, 95%CI:0.46–3.123, *P* = 0.683) (Fig. 2D). There were no COVID-19-related deaths during follow-up.

**Fig. 4:**
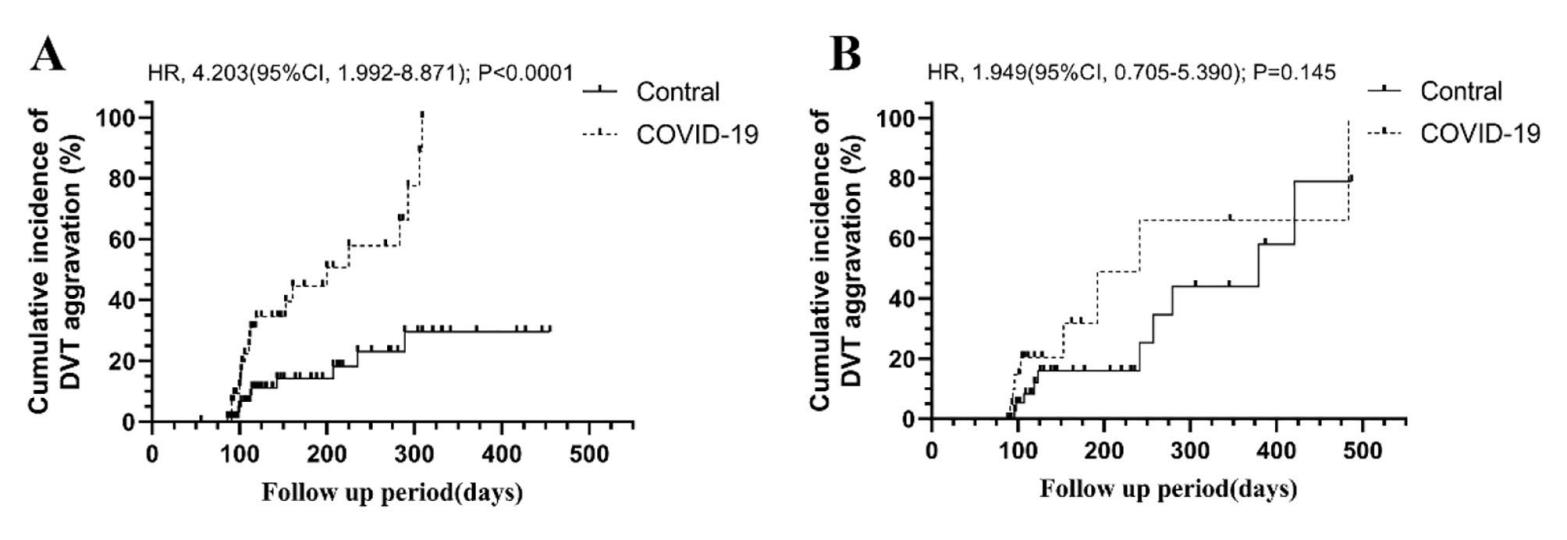
Cumulative incidence of DVT aggravation in different types of DVT in the PSM cohorts. **(A)** The cumulative incidence of DVT aggravation among patients with distal; **(B)** The cumulative incidence of DVT aggravation among patients with proximal DVT

Among patients with distal DVT (unilateral or bilateral), the COVID-19 group exhibited a significantly higher incidence rate of DVT recurrence during follow-up compared to the control group (21/44 :48% vs. 10/70 :14%, HR:4.203, 95%CI: 1.992–8.871, *P* < 0.0001) (Fig. 4A). There was no significant difference in anticoagulation duration between two groups (Three-month/Extended-term: 32/12 (COVID-19) VS 44/26 (Control), *P* = 0.313). Among patients with proximal DVT (unilateral or bilateral), the risk of DVT recurrence was similar between the COVID-19 group and the control group (HR: 1.949, 95%CI: 0.705–5.390, *P* = 0.145) (Fig. 4B). Additionally, there was also no significant difference in anticoagulation duration between two groups (Three-month/Extended-term: 10/14 (COVID-19) VS 25/17 (Control), *P* = 0.204).

**Fig. 5:**
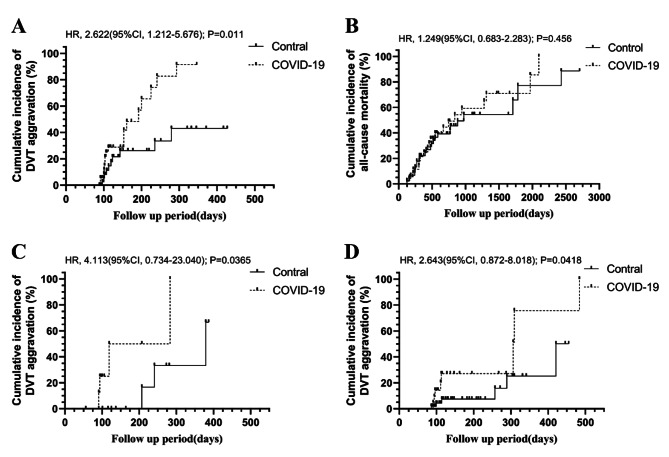
Cumulative incidence of DVT aggravation or mortality among patients with different diseases in the PSM cohorts. **(A)** The cumulative incidence of DVT aggravation among patients with advanced lung cancer; **(B)** The all-cause mortality in patients with advanced lung cancer and DVT; **(C)** The cumulative incidence of postoperative DVT aggravation; **(D)** The cumulative incidence of DVT aggravation in patients with benign lung diseases

In the analysis of principal diagnosis, patients in the COVID-19 group all exhibited significantly higher risks of DVT aggravation compared to those in the control group (Fig. 5). Among patients diagnosed with advanced lung cancer, COVID-19 increased the risk of DVT aggravation by 2.62 times (95%CI: 1.212–5.676, *P* = 0.011) when compared with the control group; however, it did not increase mortality risk (HR: 1.249, 95%CI: 0.683–2.283, *P* = 0.456), and there was no significant difference in the median survival time between two groups (839 VS 974 days, HR:1.249, 95%CI: 0.683–2.283, *P* = 0.456). Patients with a history of surgery in the COVID-19 group also had a significantly higher incidence rates of DVT aggravation than those in the control group (4/8, 50% VS 3/15, 20%, HR: 4.113, 95%CI: 0.734–23.040, *P* = 0.0365). Furthermore, among patients who diagnosed as benign lung diseases, the risk for DVT aggravation in COVID-19 group was 2.643 times higher than in the control group (95%CI: 0.872–8.018, *P* = 0.0418).

## Discussion

In this present study, we observed significant higher incidence rate of DVT aggravation in 2022 compared to 2021 among patients who had completed standard anticoagulant therapy and had at least 3 months of follow-up. Given all enrolled patients had received the same treatment strategy following guideline recommendations during these two years, we further explored the related risk factors for DVT aggravation, and history of COVID-19, increased RBC, and reduced PT were identified as risk factors for DVT aggravation in this study. However, only the history of COVID-19 infection showed a significant difference between 2021 and 2022. Therefore, we considered that a higher number of patients exposed to COVID-19 in 2022 compared to 2021 was the most important risk factor for DVT aggravation during anticoagulant therapy in our study. Previous studies have demonstrated an increased risk of first-time DVT six months after COVID-19; furthermore, the findings of this study suggest that COVID-19 is also an important risk factor for poor prognosis of DVT during treatment [12, 13, 18]. Regular DUS follow up during anticoagulant therapy was a good way to early detection of DVT aggravation [22–24].

In terms of the association between anticoagulation duration and prognosis of DVT, this study demonstrated that exposure to COVID-19 increases the risk of DVT aggravation among patients who have completed only a 3-month course of anticoagulant treatment, while no such effect is observed in patients receiving extended-term anticoagulant therapy. The recommendations regarding extended-term anticoagulant therapy administration appear to vary across guidelines [25–28]. Most guidelines suggest that the duration of anticoagulation should be determined based on an assessment of VTE risk factors, including advanced malignant tumors, paralysis, thrombophilia, and etc [20, 29]. Transient risk factors for DVT, such as inflammatory diseases and surgical history, typically warrant short-term anticoagulation until their removal. For patients diagnosed with COVID-19, some previous studies have also demonstrated that long term anticoagulation treatment does not seem to provide protection against DVT [13, 30]. However, our findings indicate that if exposure to COVID-19 occurs within 6 months before or after the diagnosis of DVT, prolonging the duration of anticoagulation treatment is necessary to ensure its effectiveness [31].

Regarding the types of DVT, there was a division regarding the impact of COVID-19 on DVT prognosis. Our findings indicate that exposure to COVID-19 increases the risk of DVT aggravation in patients with distal DVT (unilateral or bilateral). Distal DVT typically presents with fewer clinical manifestations and a lower risk of PE compared to proximal DVT, which may lead to delayed or irregular follow-up assessments for patients with distal DVT [32]. In our study, there was indeed more patients received extended-term anticoagulant therapy in patients with proximal DVT (31/66, 47%) compared to patients with distal DVT (38/114, 33%). The results highlight efficacy assessment of distal DVT has been neglected in clinical practice [1]. Therefore, it is crucial to pay attention to the adverse effects of COVID-19 on different types of DVT and particularly strengthen treatment evaluation for patients with distal DVT who have been exposed to COVID-19.

It has been previously reported that advanced lung cancer, thoracic surgery, and benign lung diseases requiring continuous anti-inflammatory treatment are all established risk factors for DVT. We also observed exposure to COVID-19 was associated with an increased incidence rate of DVT aggravation during anticoagulant therapy. Even allowing for the impact of different principal diagnoses on initial DVT diagnosis, exposure to COVID-19 will potentially affect the effect of anticoagulant therapy. This requires us not only to consider the currently principal diagnosis, but also to take COVID-19 into consideration when arranging anticoagulant therapy.

Numerous risk scoring tools have been proposed for the practical clinical assessment of DVT. Despite the widespread use of Paudua score, Caprini score, and Khorana score, several studies have reported their limited reliability in predicting DVT due to incomplete inclusion of relevant risk factors [5, 33–35]. Previous research has highlighted COVID-19 as an additional risk factor for DVT [12, 13, 18]. Our study further demonstrates that COVID-19 increases the incidence rate of DVT aggravation during anticoagulation treatment. Therefore, we should give full consideration to COVID-19 in DVT evaluation and follow-up assessments, and there is an urgent need to establish a risk scoring system and treatment strategy for patients with both DVT and exposure to COVID-19, particularly within the context of long COVID-19 and potential pandemic in the future [15, 18].

There were certain limitations in our study. Considering the retrospective nature of the study, potential selection bias and data incompleteness may impact the accuracy of our findings. As a single-center retrospective cohort study, the results might be influenced by specific practices in our hospital, and should be enhanced through future multi-center study. Furthermore, information from clinical database and telephone follow-ups might probably overrate patients’ compliance, and the prognosis of DVT was directly influenced by the normalization of anticoagulation treatment, and patients’ preference [2, 29, 32]. Considering the poor compliance with follow-up CTA among patients with PE and the missing follow-up data of PE, the correlation analysis of PE with proximal DVT was not performed. The impact of COVID-19 on prognosis of DVT during anticoagulation s needs further validation through prospective randomized clinical trials.

## Conclusions

This retrospective cohort study represents the first evidence of COVID-19’s impact on the prognosis of DVT during anticoagulation treatment in a real-world setting. Exposure to COVID-19 was associated with the high rate of DVT aggravation. Meanwhile, COVID-19 has been observed to have adverse effect on the prognosis of DVT in various respiratory benign and malignant conditions. Once exposure to COVID-19 is confirmed, patients with distal DVT should receive appropriate anticoagulant therapy and regular follow up to prevent DVT aggravation. Moreover, extended-term anticoagulant therapy has been identified as an effective approach for improving the control rate of DVT among patients with COVID-19, but further investigations are warranted to determine the optimal extended period of anticoagulant therapy in the future.

## Data Availability

The datasets used and/or analysed during the current study are available from the corresponding author on reasonable request.

## Abbreviations

COVID-19: Coronavirus disease 2019
BMI: body mass index
DBP: diastolic blood pressure
DVT: deep vein thrombosis
PE: pulmonary embolism
SCC: squamous-cell carcinoma
SCLC: small cell lung cancer
IVCT: intravenous chemotherapy
PD1: programmeddeath-1
TKI: tyrosine kinase inhibitor
RT: radiotherapy
HREZ: isonicotinic acid hydrazide, rifampicin, ethambutol, and pyrazinamide
RBC: red blood cell count
WBC: white blood cell count
PLT: blood platelet count
PT: prothrombin time
